# Immunotherapy monotherapy for patients with recurrent nonmetastatic larynx cancer who refuse salvage total laryngectomy

**DOI:** 10.3389/fonc.2025.1626880

**Published:** 2025-11-17

**Authors:** Courtney Brooke Shires, Merry E. Sebelik

**Affiliations:** 1 West Cancer Center, Germantown, TN, United States; 2 Department of Otolaryngology, Emory University, Atlanta, GA, United States

**Keywords:** larynx cancer, head and neck cancer, squamous cell carcinoma, cervical metastasis, recurrence, immunotherapy, prognosis, laryngectomy

## Abstract

**Objectives/Hypothesis:**

Total laryngectomy has traditionally been recommended for patients with recurrent larynx cancer after radiation or chemoradiation. Some patients refuse salvage surgery. Historically, these patients have been placed on hospice or palliative chemotherapy. Immunotherapy has recently added another treatment modality to our armamentarium.

**Methods:**

8 patients with recurrent larynx cancer declined salvage laryngectomy. They were started on immunotherapy alone. We recorded their demographics, initial cancer stage, initial cancer treatment, combined positive score (CPS) values, need for tracheostomy, addition of chemotherapy to their immunotherapy, and response to therapy.

**Results:**

62.5% were African American, while 37.5% were Caucasian. Most had early (stage 1 or 2 disease) at the time of initial diagnosis. 75% had radiation alone as their initial treatment, while 25% had chemoradiation. All of these patients had high CPS scores. 37.5% progressed on 4 rounds of immunotherapy and are deceased. Chemotherapy was added to the regimen of 50% of the patients after poor response to immunotherapy. 75% of these patients are all still alive after 1 year of treatment. 75% of all patients needed tracheostomy while on salvage treatment. One patient (12.5%) has had a long-lasting positive response to immunotherapy alone.

**Conclusions:**

Our patients with nonmetastatic recurrent larynx cancer were found to have high CPS scores, which suggests favorable response to immunotherapy. Most patients with recurrent larynx cancer on immunotherapy required a tracheostomy. These patients had poor response on immunotherapy alone, but had prolonged survival with added chemotherapy. Salvage laryngectomy is the only curative option for these patients, but for those patients that refuse surgery, chemotherapy with immunotherapy has better results than immunotherapy alone. Our results reveal a possible clinical phenomenon, which needs to be confirmed by large sample studies.

## 1 Background

Despite advancements in treatments for head and neck mucosal squamous cell carcinoma (HNMSCC), the overall 5-year survival of head and neck cancer has not changed significantly in the last few decades and still hovers between 40–50% (1). This is primarily due to high recurrence rates, aggressive growth, and propensity for metastasis. 70% of patients with HNMSCC are diagnosed at an advanced stage. About half of patients relapse after curative treatment or become refractory to therapy. Before the advent of immunotherapy, 1-year survival in these patients was only 17% to 37% and median survival time was 5 to 10 months (2).

Laryngeal squamous cell carcinoma, as part of HNMSCC, has been relatively understudied and is often grouped together with other types of HNMSCC (i.e., hypopharynx and oropharynx cancer). However, due to its unique anatomical location, this subtype requires more targeted research and analysis (3).

Salvage laryngectomy is a common treatment for recurrent larynx cancer after nonsurgical treatment. Contrera evaluated patterns of failure following salvage head and neck surgery. He retrospectively reviewed 280 patients who underwent salvage surgery for recurrent mucosal squamous cell carcinoma from 1997 to 2018. The 2- and 5-year cumulative incidence rates of second recurrence were 48.3% and 54.9%. At 5 years, second locoregional recurrence was twice as common as distant recurrence (4). When we offer salvage surgery to recurrent larynx cancer patients, we must be truthful that their chance of another recurrence after laryngectomy is approximately 50%. We discussed established recurrent rates after surgery with our patients, and 8 patients with recurrent localized larynx cancer subsequently declined salvage laryngectomy.

Historically, these patients that have declined surgical salvage after failure with radiation or chemoradiation have been placed on hospice or palliative chemotherapy. Immunotherapy has recently added another treatment modality to our armamentarium.

The arrival of immune checkpoint inhibitors (ICIs) has opened up new treatment avenues for head and neck cancer, improving one-year survival rates in patients with recurrent metastases to 36% to 57% and median survival to 7.7 to 13.0 months (5, 6). Recently, many efforts have been made to develop ICI-based therapies for not only recurrent metastasis but also locally advanced cases (like in our cohort), and attention has focused on not only anti-PD-1 antibody agents but also anti-PD-L1 and anti-CTLA-4 antibody agents (2).

Much data on ICIs in metastatic head and neck cancer patients has become available. The response rates remain low (13%-20%). Alsavaf found that smoking status, marijuana use, and alcohol consumption did not have a statistically significant impact on overall survival (OS) in patients with recurrent or metastatic HNMSCC treated with ICIs (7).

There are many studies regarding neoadjuvant immunotherapy for hypopharynx and larynx cancer. We wanted to evaluate immunotherapy in a salvage, palliative setting of nonmetastatic recurrent larynx cancer in patients that declined potentially curative salvage surgery (8).

## 2 Methods

8 patients with recurrent larynx cancer (after radiation with or without chemotherapy) and no metastasis were seen in our clinic. These patients were evaluated in a multidisciplinary clinic and were presented at our head and neck tumor board meeting. Our patients did not qualify for salvage partial laryngectomy due to size or location or tumor, preoperative swallowing dysfunction, or lung disease. We informed our patients that salvage total laryngectomy (TL) is the standard of care for treating recurrent larynx cancer after radiation +/-chemotherapy and was recommended for them. We discussed that even after TL, there continues to be a significant recurrence risk for their cancer. However, we discussed that TL also offers a potentially curative intent, while systemic therapy (chemotherapy or immunotherapy) is only considered palliative. Patients were told that if they declined surgery, that our goals would shift to disease control and symptom management rather than cure. All patients met with our Speech Therapist to discuss expected postoperative outcomes.

After consideration, all of our included patients declined salvage TL. Reasons for refusal included concerns about functional outcomes, postoperative quality of life, their ability to tolerate a surgery due to medical comorbidities, or the possibility of recurrence after this potentially curative surgery.

Staging was confirmed with PET scan. The patients were started on single agent immunotherapy (pembrolizumab). If progression was seen after 3–4 rounds of immunotherapy, chemotherapy (carboplatin and 5 fluorouracil) was added to immunotherapy. We recorded patients’ demographics, initial cancer stage, initial cancer treatment, CPS values, need for tracheostomy, addition of chemotherapy to immunotherapy, median progression-free survival (PFS) for responders, common terminology criteria for adverse events (CTCAE), and response to therapy.

## 3 Results

The average age of our patient was 72 years old (**Table 1**). Of our 8 patients, 62.5% (n=5) were African American, while 37.5% (n=3) were Caucasian. Most had early (stage 1 or 2 disease) at the time of initial diagnosis. 75% (n=6) had radiation alone as their initial treatment, while 25% (n=2) had chemoradiation. All of these patients had high CPS scores (>1). 37.5% (n=3) of our patients progressed on 3–4 rounds of immunotherapy and are deceased. Chemotherapy was added to the regimen of 50% of the patients after poor response (progression of disease) on immunotherapy alone. Glottis and supraglottis were the most common location of initial disease and recurrent disease (**Table 2**).

**Table 1:** Patients with recurrent larynx cancer who received immunotherapy after refusing laryngectomy.

Patient	Race/sex	Age	Previous XRT	Trach	Initial stage	Chemo with immuno?	CPS	Status	Time after immuno started that chemo was added	Chemo agent	Progression-free survival for responders (after starting chemo)	CTCAE
A	AAM	81	yes	yes	T3N1M0	no	32	deceased after 9 months	NA	NA	NA	Grade 1
B	WM	86	yes	yes	T1bN0M0	no	17	deceased after 11 months	NA	NA	NA	Grade 2
C	WM	66	yes	yes	T2N0M0	no	21	deceased after 12 months	NA	NA	NA	NA
D	AAM	54	yes	yes	T2N0M0	yes	9	living after 13 months	12 weeks	carboplatin, 5 fluorouracil	10 months	Grade 1
E	AAM	72	yes	yes	T1N0M0	yes	14	deceased after 15 months	10 weeks	carboplatin, 5 fluorouracil	NA	NA
F	AAF	71	yes + chemo	yes	T2N1M0	yes	24	living after 15 months	11 weeks	carboplatin, 5 fluorouracil	12 months	Grade 1
G	WM	84	yes	no	T2N0M0	yes	16	living after 12 months	13 weeks	carboplatin, 5 fluorouracil	9 months	NA
H	AAF	65	yes + chemo	no	T3N0M0	no	6	living after 20 months	NA	NA	17 months	NA

**Table 2:** Subsites of laryngeal cancer at initial diagnosis and recurrence.

Patient	Subsite of initial larynx cancer	Subsite of recurrent larynx cancer
A	glottis and supraglottis	glottis (anterior commissure and thyroid cartilage)
B	glottis	glottis and supraglottis
C	supraglottis	supraglottis
D	subglottis	sugblottis and glottis
E	supraglottis	supraglottis
F	supraglottis	supraglottis
G	glottis and supraglottis	glottis (anterior commissure and thyroid cartilage)
H	glottis and supraglottis	supraglottis and glottis

75% (n=6) of the patients on immunotherapy with chemotherapy have been living 12–15 months after initiating salvage treatment. Median PFS for responders after combined chemotherapy and radiation was 10.3 months.

One patient (12.5%) has had a long-lasting positive response to immunotherapy alone with nearly no measurable tumor on imaging. She has been on immunotherapy for 20 months with no progression of disease.

75% (n=6) of our patients needed tracheostomy while on salvage treatment.

There were no Grade 3, 4, or 5 common terminology criteria for adverse events (CTCAE). 37.5% (n=3) of patients had grade 1 CTCAE, and one patient (12.5%) had a grade 2 CTCAE. Half of the CTCAE were in patients that received only chemotherapy, and half of the patients that experienced CTCAE received chemotherapy and immunotherapy.

## 4 Discussion

### 4.1 Data on immunotherapy for head and neck cancer

There are many immunotherapy-related clinical trials of various designs for HNSCC. There have been several negative trials (CheckMate651 NCT02741570; EAGLE NCT02369874, KESTREL NCT02551159, KEYNOTE-669 NCT03358472) and several discontinued trials (INTERLINK-1 NCT04590963, LEAP-010 NCT04199104, KEYNOTE-669 NCT03358472, INDUCE-3 NCT04128696, INDUCE-4 NCT04428333, JAVELIN H&N 100 NCT02952586. Immunotherapy has a long history of research and a variety of mechanisms of action have been developed. Nevertheless, the number of truly useful therapies remains limited (2). We will discuss the trials and studies that have included immunotherapy for use in head and neck.

#### 4.1.1 Palliative immunotherapy

The CheckMate-141 study (NCT02105636) was a phase III trial comparing nivolumab monotherapy as second-line therapy with investigator’s choice of either cetuximab, docetaxel or methotrexate in patients with recurrent metastatic head and neck mucosal squamous cell carcinoma (R/M-HNMSCC) resistant to platinum drugs. The primary endpoint, overall survival (OS), demonstrated the superiority of nivolumab monotherapy (median 7.5 months) over investigator’s choice of therapy (median 5.1 months) (5).

The KEYNOTE-040 study (NCT02252042) was a phase III trial comparing pembrolizumab monotherapy with investigator’s choice of one of cetuximab, docetaxel or methotrexate as second-line therapy in patients with R/M-HNMSCC refractory to platinum drugs. The primary endpoint of OS failed to demonstrate the superiority of pembrolizumab monotherapy over investigator’s choice of therapy. However, an additional analysis of all survival data showed the superiority of pembrolizumab (median 8.4 months) over standard therapy (median 6.9 months) (10). These still are not notable differences.

The HAWK study (NCT02207530) was a phase II trial evaluating the efficacy and safety of single-agent anti-PD-L1 antibody drug durvalumab in platinum-resistant R/M-HNSCC patients with high PD-L1 expression (≥25%). The primary endpoint of ORR (objective response rate) was only16.2% (2, 11).

Although ICIs have demonstrated efficacy in many clinical trials by acting through different mechanisms to those of conventional chemotherapy (cytotoxic agents and molecular-targeted agents), response rate to monotherapy is only 10–30%, and only a small fraction of these patients achieve long-term survival (2). Our results with pembrolizumab in 8 recurrent larynx cancer patients also showed a low rate of response. Our subgroup does not have any better response rate in patients with only local disease and no distant metastasis.

As the response rates to ICI remain low (13%-20%) in metastatic head and neck cancer patients, Alsavaf’s group evaluated other factors such as smoking status, marijuana use, and alcohol consumption, and found that these did not have a statistically significant impact on OS in patients with recurrent or metastatic HNSCC treated with ICI (7). All of our patients smoked tobacco, so this also did not play a role in outcomes.

Only 13% of the patients in Alsavaf’s study had larynx cancer, revealing that there is not a large study on the recurrent nonmetastatic larynx cancer population (7). This is similar to our study in low numbers.

#### 4.1.2 Palliative immunotherapy with chemotherapy

Fang’s group looked at PDL1 inhibitors and taxel and cisplatin for recurrent larynx/hypopharynx cancer. His retrospective study examined the efficacy and survival outcomes of PD-1 inhibitors combined with paclitaxel and cisplatin regimen in the treatment of recurrent and metastatic hypopharyngeal/laryngeal squamous cell carcinoma (RMHSCC/RMLSCC). All patients received PD-1 inhibitors combined with albumin-bound paclitaxel (260mg/m^2^) and cisplatin (60mg/m^2^) for 3–4 cycles. Fifty patients with RMHSCC/RMLSCC had an objective response rate (ORR) of 56.0% (28/50). The 1-year and 2-year OS rates were 80.2% and 68.6%, respectively, while the 1-year and 2-year progression free survival (PFS) rates were 44.7% and 26.0%. They concluded that in the treatment of RMHSCC/RMLSCC with paclitaxel and cisplatin + PD-1 inhibitors, survival rates of patients can be improved while ensuring the safety of the treatment regimen. They had a short follow up period of up to 2 years (11). This was similar to our study where we showed that when chemotherapy was added to immunotherapy, survival increased.

This positive result with a combination of treatment with PD-1 inhibitor + paclitaxel and cisplatin in RMHSS/RMLSCC of hypopharynx/larynx cancer, was then carried over by Fang’s group to another set of patients. He achieved a 94.1% response rate in neoadjuvant treatment of hypopharyngeal/laryngeal cancer (12).

There were some differences in Fang’s group than ours. He excluded patients over 70 years old, while 63% of our patients were over 70 years old. 60% of our elderly patients are doing well on the combination of immunotherapy and chemotherapy. Fang included some patients who had not had previous treatment, so radiation was still an option for these patients. All of our patients had previous radiation. Fang included hypopharynx cancer in the cohort as well. Only a small portion of their patients had recurrent larynx cancer after radiation (12). Our patients had recurrent cancer of the larynx after radiation with no distant metastasis.

This combination of chemotherapy and immunotherapy was beneficial in our patients, with these patients having the longest survival. Our study used immunotherapy as palliative, not in a neoadjuvant manner as previous studies. Immunotherapy in our study was used along with chemotherapy to give better results, as immunotherapy alone in a palliative setting for recurrent localized larynx cancer had limited efficacy. Adding chemotherapy to immunotherapy can lead to increased survival rates in various cancers due to several synergistic mechanisms that enhance the overall anti-cancer effect. However, despite promising signals, systemic treatment cannot replace surgery when curative salvage is possible. Chemo-immunotherapy should not replace the standard of care for recurrent larynx cancer after radiation (TL), but instead be used as a palliative option over chemotherapy alone or immunotherapy alone in patients that refuse total laryngectomy.

There were no Grade 3, 4, or 5 CTCAE among our patients. There was no increase in severe CTCAE in the patients that received chemotherapy and immunotherapy versus those that received immunotherapy alone.

#### 4.1.3 Neoadjuvant immunotherapy

In addition to Fang, a few other groups have looked at immunotherapy + chemotherapy in the neoadjuvant setting for HNMSCC. They wanted to investigate the clinical efficacy, preservation of laryngeal function, and safety differences between PD-1 inhibitors combined with chemotherapy, and targeted therapy combined with chemotherapy in locally advanced hypopharyngeal squamous cell carcinoma patients. PD-1 inhibitors combined with chemotherapy showed better short-term efficacy compared to targeted therapy. Additionally, a trend toward improved long-term survival was observed with PD-1 inhibitors but not with targeted therapy. Results for both groups indicate that neoadjuvant therapy is both safe and manageable (13).

A second group look at neoadjuvant in hypopharynx. Neoadjuvant therapy of PD-1 inhibitor combined with paclitaxel and cisplatin effectively improved the major pathological response (MPR) and laryngeal preservation surgery (LPS) rates of locally advanced hypopharynx cancer patients, especially in those at clinical stage IV. The 1-year and 2-year PFS rates were 97.1% and 93.8% for all patients, with stage IV patients having a 1-year PFS of 92.2%. They had promising results (14).

The most recent study to gain accolades is the phase III KEYNOTE-689 trial presented at the American Society of Clinical Oncology (ASCO) meeting in 2025. Results of using perioperative/neoadjuvant pembrolizumab in patients with locally advanced head and neck squamous cell carcinoma are promising. The trial showed a statistically significant improvement in event-free survival for patients treated with perioperative pembrolizumab plus standard of care (SOC) compared to SOC alone. Patients receiving pembrolizumab were also more likely to achieve a major pathologic response (≥90% tumor reduction) (9).

### 4.2 Immunotherapy in our patients

Salvage surgery was discussed multiple times with our patients. We revisited the topic of laryngectomy when the patients did not seem to have a measurable response to medical therapy either clinically or on imaging. The patients persistently declined laryngectomy. 75% of our patients developed dyspnea while on systemic therapy, requiring tracheostomy. It was interesting that the patients chose continued systemic therapy for organ preservation and quality-of-life considerations, but still agreed to tracheostomy. Our patient with the durable response on immunotherapy alone has been able to avoid tracheostomy thus far.

This is a challenging subset of patients, as there is a stigma associated with TL. An extended discussion should be had regarding the potential for speech and swallowing rehabilitation to calm fears regarding surgery. The patients need to understand that there is a great possibility of returning to oral feeds and other forms of speech after TL. For patients that refuse laryngectomy or patients that cannot undergo surgery due to age or medical comorbidities, they need to understand that immunotherapy alone is not an acceptable alternative to extend their life or prevent disease progression. If TL is refused, adding chemotherapy to immunotherapy can slow disease progression and extend life.

While we have noted a trend of our findings, we realize that our report has a notable shortcoming. Our cohort is very small (n=8), limiting the statistical power and generalizability of the findings. Conclusions from our small sample size must be interpreted cautiously.

We hope to take the results of these few patients and continue collecting a larger group of patients in order to produce relatable statistics.

In the future, we would like to examine more detailed data from this subset of recurrent laryngeal cancer patients declining salvage surgery. We would like to evaluate whether tumors of different laryngeal subsites (glottis versus subglottis versus supraglottis) have better outcomes with immunotherapy. We need to evaluate whether those with higher CPS scores have better results than those with lower CPS scores. Do tumor volumes at the initiation of immunotherapy affect outcome? This content would add knowledge that could be relayed to patients in this situation.

## 5 Conclusions

Our patients with nonmetastatic recurrent larynx cancer were found to have high CPS scores, which suggests favorable response to immunotherapy. Most patients with recurrent larynx cancer on immunotherapy required a tracheostomy. These patients had poor response on immunotherapy alone, but had prolonged survival with added chemotherapy. Salvage laryngectomy is the only curative option for these patients, but for those patients that refuse surgery, chemotherapy with immunotherapy has better results than immunotherapy alone. Our results reveal a possible clinical phenomenon, which needs to be confirmed by large sample studies.

## Data Availability

The original contributions presented in the study are included in the article/supplementary material. Further inquiries can be directed to the corresponding author.
